# Treatment outcome in adolescent and young adult acute lymphoblastic leukaemia (ALL) on BFM-95 protocol: experience of a tertiary care Institute from North India

**DOI:** 10.3332/ecancer.2025.1962

**Published:** 2025-08-07

**Authors:** Alpika Shukla, Shailendra Prasad Verma, Anil Kumar Tripathi, Swasti Sinha

**Affiliations:** Clinical Hematology and BMT, KGMU, Lucknow 226003, India

**Keywords:** acute lymphoblastic leukaemia, adolescent and young adult, BFM-95

## Abstract

**Background:**

BFM-95 protocol is among the common regimens used to treat adolescent and young adult (AYA) acute lymphoblastic leukaemia. Five-year survival in AYA patients with acute lymphoblastic leukaemia is inferior when compared to children.

**Objective:**

To study treatment outcomes in adolescent and young adult patients with acute lymphoblastic leukaemia on BFM 95 protocol.

**Material and Methods:**

We retrospectively analysed the available data of 75 patients diagnosed with acute lymphoblastic leukaemia in the AYA age group who received treatment as per BFM-95 protocol from 2016 to 2020 in the clinical hematology department in a tertiary health care centre.

**Results:**

Among 75 patients, 56 were male and 19 were female. High-risk patients were 15 (20%), 3 due to poor prednisolone response and 12 due to high-risk cytogenetics. Most of the patients were CNS-1 and three patients were of CNS-3. Eight (10.6%) patients did not complete induction treatment. The median duration of induction phase A was 40 days (range 35–45 days). The most common complication during induction phase A treatment was febrile neutropenia which was seen in 21 patients (28%), followed by transaminitis in 12 patients (21.1%). The median duration of follow-up was 2 years (range 8 months–5 years). Five (6%) patients’ bone marrow were not in remission after induction-A. Relapse occurred in 23 patients (30.6%), with the highest incidence observed during the maintenance phase of treatment. Thirty-three (44%) patients completed maintenance and were still on follow-up. Twenty-three (30.7%) patients relapsed while six patients were lost to follow-up during maintenance. At a median follow-up of 2 years, disease-free survival was (44%).

**Conclusion:**

This study on AYA patients with acute lymphoblastic leukaemia/lymphoma using a pediatric protocol in a resource-limited setting observed suboptimal survival rates, which may be attributed to the retrospective design, significant data gaps and a small patient cohort with limited follow-up.

## Introduction

Adolescents and young adults (AYA) make up approximately 25% of all cases of acute lymphoblastic leukaemia/lymphoma (ALL) [1]. The age cut-off for AYA patients ranges from 15 years to 39 years in various studies [2]. Treatment outcomes on the pediatric-based protocol for this age group have generally been poor as compared to children due to the additional toxicity of treatments in this age group and the increased incidence of high-risk cytogenetics compared to children [3]. However, AYA patients do better on pediatric-inspired ALL protocols compared to adult-inspired chemotherapy protocols.

The reported 3-year disease-free survival in AYA patients with pediatric protocol versus adult protocol is 72.2% and 44.6%, respectively, whereas overall survival is 75.3% versus 64.1%, respectively [4]. This situation underscores the necessity for customised strategies that address the unique biological and logistical challenges faced by the AYA population in resource-constrained regions.

In this paper, we report outcomes with a modified BFM 95 protocol in a group of patients aged 15–25 years, the efficacy and toxicity profile of drugs used in this protocol and the influence of various prognostic factors on the survival of these patients.

## Methods

This retrospective study included data from patients aged 15–25 years who were newly diagnosed with Acute Lymphoblastic leukaemia/Lymphoma (B-ALL/T-ALL), between 2015 and 2020 treated in the clinical hematology department. Data were obtained from departmental medical records. We aimed to assess event-free survival (EFS), with secondary focuses on examining how toxicity and unfavourable risk factors influenced overall survival.

Event-free survival was defined as the duration from enrollment to any occurrence, such as treatment failure, relapse or death. Kaplan–Meier analysis was used to estimate event-free survival rates.

### Relapse

Defined as recurrence of disease (as the reappearance of blasts in the bone marrow or peripheral blood (medullary relapse) or the presence of blasts in the cerebrospinal fluid (CSF), testicular involvement or involvement of other extramedullary sites) post remission [5]. Patients who experienced relapse were switched to alternative treatment protocols.

**TRANSAMINITIS** – SGOT/SGPT values more than three times normal were used as cut-off for transaminitis.

### Diagnosis

The diagnosis was confirmed through flow cytometry on peripheral blood or bone marrow samples. Additionally, CSF cytomorphology was performed, along with a molecular panel based on multiplex RTPCR (panel including BCR-ABL, ETV6/RUNX1, E2A/PBX1 and MLL/AF4) and cytogenetic analysis on the bone marrow samples.

Patients were categorised into three risk groups based on their clinical and genetic profiles as per BFM 95 protocol (see Supplementary section).

Modified BFM 95 protocol was used with the following adjustments:

1. **L-asparaginase**: Native *Escherichia coli* L-asparaginase, dose of 10,000 U/m^2^ through a 1-hour intravenous infusion. L-asparaginase was omitted in Ph +ve patients receiving tyrosine kinase inhibitor.
2. **High-dose methotrexate (HDMTX)**: In the extra compartment M phase, HDMTX was given at a dose of 5 gm/m^2^, followed by rescue with leucovorin.
3. **Cranial radiation therapy (RT)**: In the reintensification phase B, high-risk patients received 18 Gy, while standard-risk patients received 12 Gy [4].
4. **Intrathecal methotrexate**: Administered at a dose of 12 mg during induction phase 1A, phase 1B, Extra compartment Phase M and reintensification phase 1B.
5. **Dasatinib**: Ph-positive patients were treated with dasatinib tablets, 100 mg once daily.

## Results

### Baseline parameters

**Figure 2.**
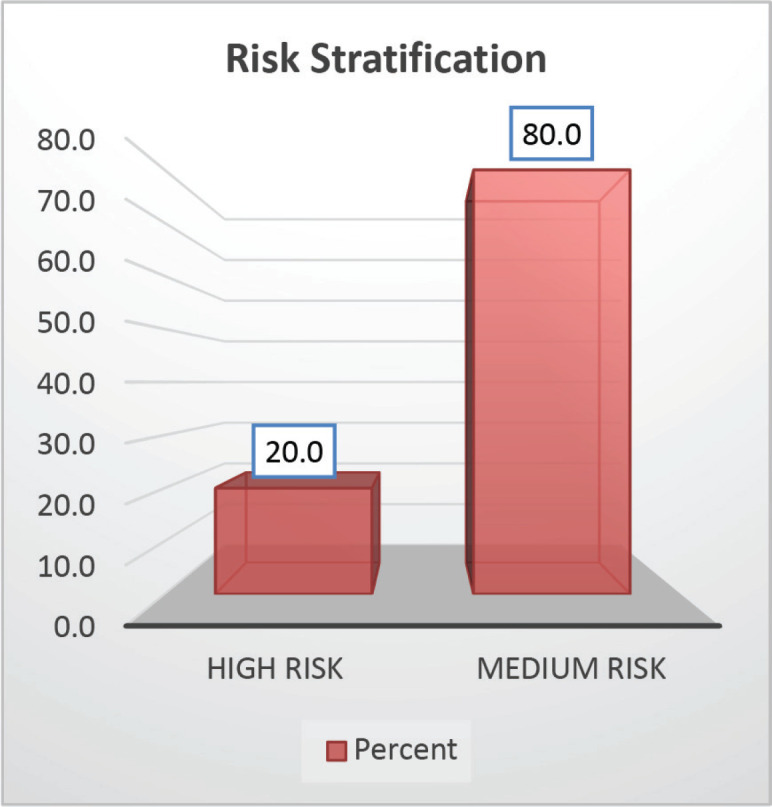
Risk wise stratification.

**Figure 3.**
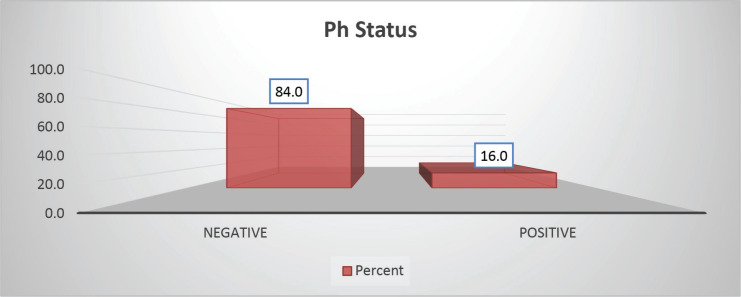
Ph status-wise distribution.

**Figure 4.**
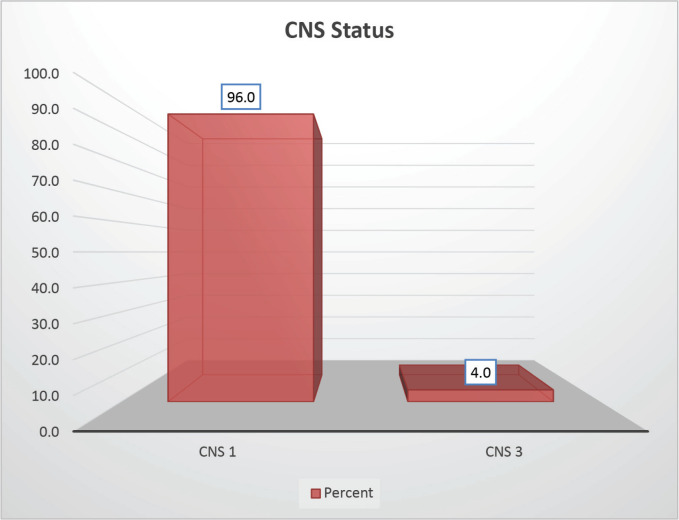
CNS status-wise distribution.

**Figure 5.**
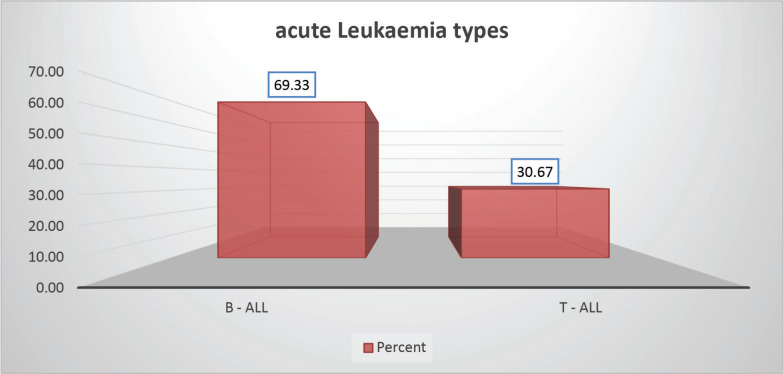
Leukaemia type-wise distribution.

The cohort primarily consisted of 56 (74.67%) male patients and 19 (25.33%) females. In terms of risk stratification, the majority (80%) were in the intermediate-risk category, while 20% were classified as high risk (Figure 2). Most individuals (84%) were PH-negative, indicating that only 16% tested positive for this genetic marker (Figure 3). Ninety-six percent of patients were classified as CNS 1, meaning no central nervous system (CNS) involvement and 4% had CNS 3 status, indicating significant CNS involvement (Table 1) (Figure 4). Regarding the type of leukaemia, a large proportion (69.33%) of patients had B-cell Acute Lymphoblastic leukaemia (B-ALL), while (30.67%) had T-cell Acute Lymphoblastic leukaemia (T-ALL) (Figure 5).

### Complications and toxicities

**Figure 6.**
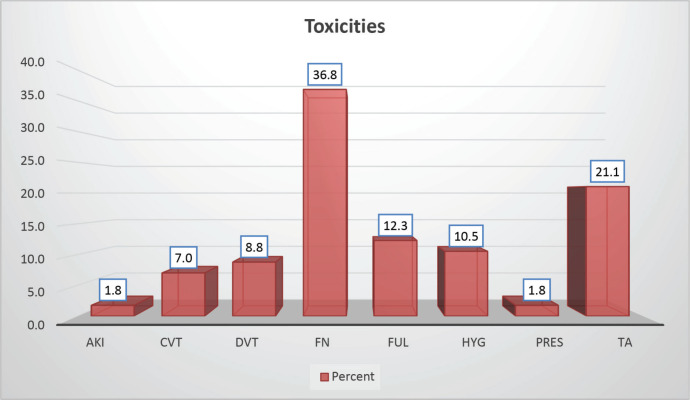
Distribution according to toxicity profile of study population.

**Figure 7.**
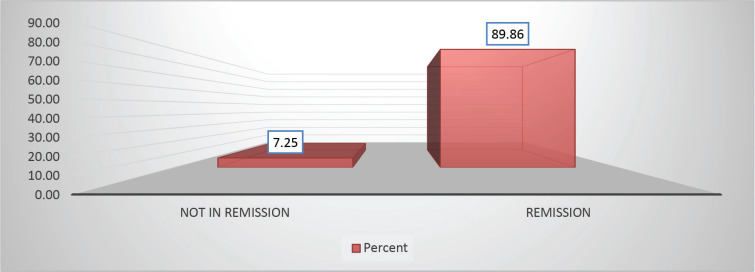
Distribution based on remission versus not in remission A (89.86% versus 7.25%) of study population post Berlin-Frankfurt-Münster (BFM) Induction 1A.

Throughout the various phases of treatment, several complications were encountered. During the induction A phase, the predominant issue was febrile neutropenia, affecting 21 patients (36.8%), followed closely by transaminitis in 12 patients (21%) (Figure 6). Sixty-seven (89.86%) patients were in remission after induction phase A (Figure 7). Moving into induction phase B, cytopenias emerged as the primary concern, often necessitating treatment prolongation by 3–7 days. Additionally, drug-induced liver injury and vincristine-induced neuropathy were notable complications during this phase (Tables 2 and 3).

In the extracompartment M phase, mucositis of grades 1–2 and liver enzyme abnormalities due to 6-Mercaptopurine were managed conservatively, alongside occurrences of fluid overload. The reintensification phase generally saw good chemotherapy tolerance, with grades 1–2 neutropenia observed in 6 patients and steroid-induced psychosis in 2 patients. Finally, during the maintenance phase, the main challenges included 6-Mercaptopurine-induced cytopenia affecting 9 patients and deranged liver enzymes noted in 11 patients.

A statistically significant difference was not observed between patients treated under the BFM 95 protocol, regardless of whether they had T-ALL or B-ALL.

**Figure 8.**
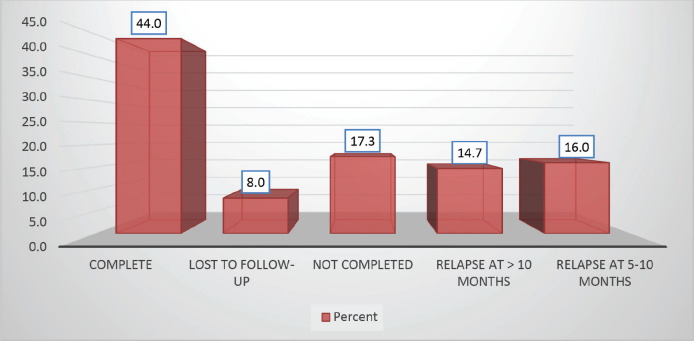
Study population distribution as per protocol completion.

**Figure 9.**
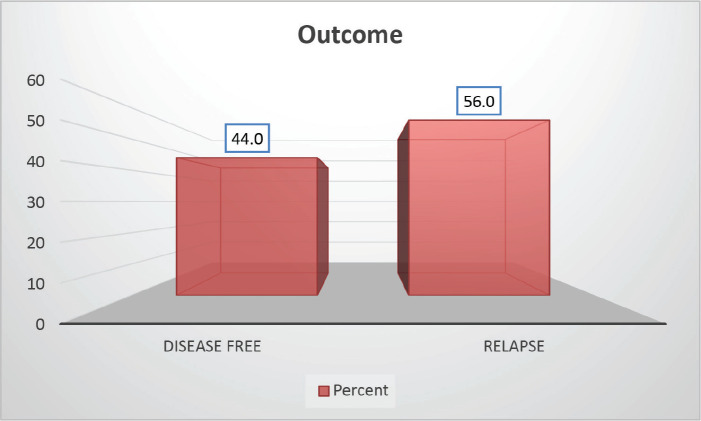
Study population distribution as per outcome.

**Figure 10.**
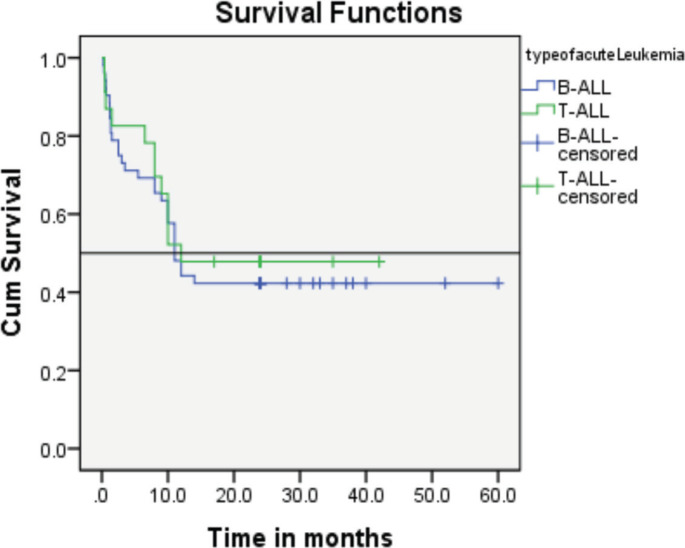
Comparison of event-free survival was between patients with T-ALL and B-ALL showing no significant difference.

**Figure 11.**
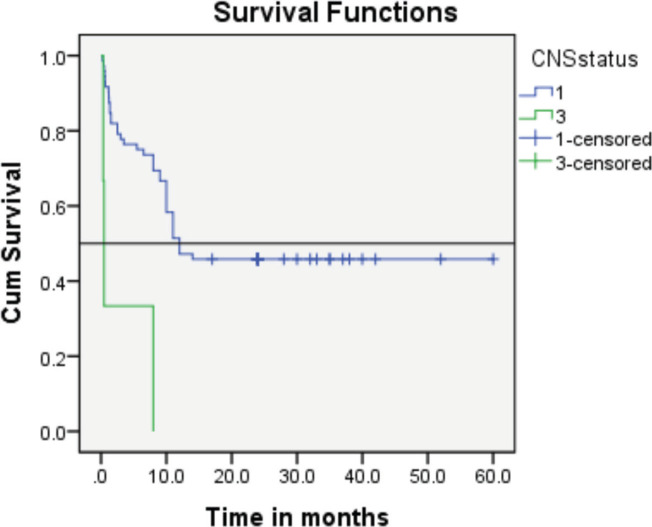
The event-free survival rate of the CNS 3 subgroup verses that of the CNS 1 subgroup.

**Figure 12.**
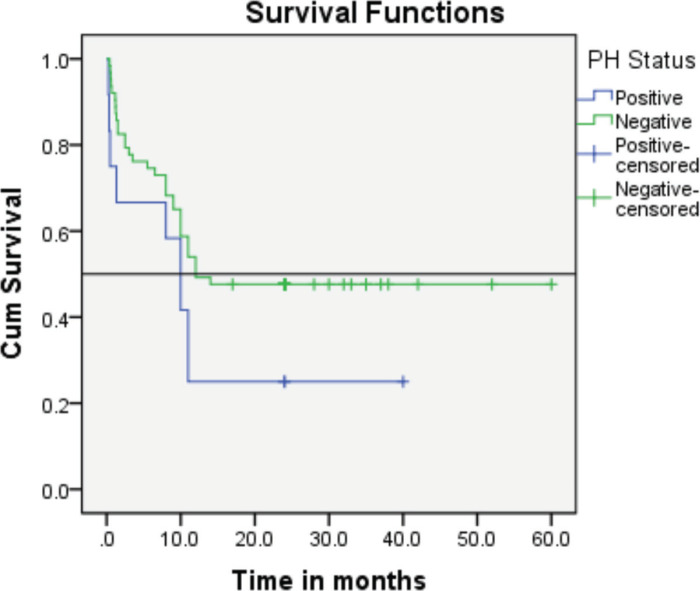
The event-free survival rate of the Ph+ subgroup verses that of the Ph-negative AYA patients (25% versus 44%).

Follow-up duration in our study ranged from 8 months to 5 years, the median follow-up was 2 years. At the time of analysis, 33 patients (44%) had successfully completed maintenance therapy and were still being monitored. Twenty-three patients experienced relapse, primarily occurring during the maintenance phase (Figures 8 and 9). Six patients were lost to follow-up during this period. Thirteen patients expired out of which treatment-related mortality was reported in five (6.66%). Patients were advised referral for bone marrow transplants in high-risk patients and patients who relapsed but none of the patients opted for same. At the median follow-up of 2 years, the disease-free survival rate was 44%. There was no significant difference in DFS between T-cell ALL and B-cell ALL (Figure 10). However, CNS involvement and Philadelphia (Ph) chromosome status were significantly associated with differences in DFS (Figures 11 and 12), underscoring their prognostic impact.

## Discussion

Treating AYA patients with ALL using pediatric-based chemotherapy regimens generally leads to better outcomes than using adult-based regimens [5, 6, 19]. Pediatric treatments include multiple doses of asparaginase, more frequent use of vincristine and steroids and intensive prevention of leukaemia in the central nervous system, along with a longer maintenance phase [7]. Particularly for patients aged 18–25, pediatric treatments have proven to be more effective, leading to more AYA patients receiving pediatric-style care [8].

One of the largest earlier studies retrospectively compared the outcome of 321 AYA patients who were treated on either the Children’s Cancer Group (CCG) trials or the adult Cancer and Leukaemia Group B (CALGB) protocol. A significantly higher 7-year EFS rate for patients treated with CCG regimens than for patients treated with CALGB regimens (63% versus 34%, *p* < 0.001) was seen [9].

Research by Goldberg *et al* [10] from the Dana Farber Cancer Institute found no significant difference in event-free survival between T-ALL and B-ALL patients, although T-ALL cases had higher rates of induction failure and CNS relapses [10].

Comparison of disease-free survival between patients with T-ALL and B-ALL in our study showed no significant difference consistant with the above study.

Retrospective analysis done by Bassan *et al* [11] found that adult patients with mature B-ALL had an 18% incidence of CNS involvement at the time of presentation, which is significantly higher than the overall incidence of 4.5% [11].

In our cohort, CNS involvement was reported in 4% of cases, aligning closely with other studies where incidence rates typically hover around 5%. The complete remission rate in our population was 89.86%, slightly lower than the rates reported in previous similar studies (>90%).

Regarding Philadelphia chromosome-positive ALL (Ph+ ALL) studies have shown poor prognosis in patients treated solely with chemotherapy, with high relapse rates and lower post-treatment survival rates compared to Ph-negative patients [12, 13].

In our cohort, the event-free survival rate of the Ph+ subgroup was notably lower than that of Ph-negative AYA patients (25% versus 44%). Among the 12 patients with Ph+ ALL, 3 (25%) achieved remission and continue to be monitored. The reason for poor survival of the ph-positive group can be attributed to frequent interruptions in the treatment schedule due to cytopenia, effusion in patients on TKI with chemotherapy, noncompliance of patients due to cytopenia and frequent dose reduction of TKI.

A study by Ribera *et al* [14, 15] showed that there were more delays during reinduction and frequent dose adjustment for vincristine and l-asparaginase while treating young adults compared to adolescents, with statistically significant differences observed (*p* = 0.04 and *p* = 0.03, respectively). Notably, in our cohort, frequent dose modifications were required for L-asparaginase, while significant treatment delays were primarily attributed to cytarabine-induced cytopenia.

Various Indian studies have also evaluated the efficacy of BFM protocol in AYA patients.

A multicentric Indian study reported 2-year event-free, relapse-free and overall survival (OS) of 64%, 75% and 75%, respectively [16].

A study from TATA Hospital Mumbai reported a 3-year EFS and OS of 59.4% and 61.8%, respectively [15] our findings indicated a 2-year event-free survival of around 44%. Lower survival rates may be attributed to higher risk subtypes such as Philadelphia positive, increased risk of infections due to intensive regimen, suboptimal supportive care and poor patient compliance.

Moreover, treatment-related complications, particularly infections, contributed significantly to lower EFS rates compared to those reported in more developed settings [17, 18]. These challenges highlight the critical need for discussions on protocol adjustments and improvements in supportive care to address the unique conditions faced by AYA patients with ALL.

AYA with ALL have demonstrated improved outcomes when treated with pediatric-inspired regimens. Among these, the modified BFM protocol has achieved high induction remission rates, reflecting the regimen's initial effectiveness. However, the 2-year EFS remains suboptimal, largely due to infection-related mortality and post-induction treatment abandonment. These challenges highlight the need for enhanced supportive care and psychosocial interventions to ensure treatment adherence and reduce morbidity in this population [19, 20].

## Conclusion

This study on AYA patients with acute lymphoblastic leukaemia/lymphoma using a BFM95 protocol in a resource-limited setting observed suboptimal survival rates, The lower survival rates observed in our study cohort may be attributed to high-risk subtypes, such as Philadelphia chromosome-positive cases, increased susceptibility to infections due to intensive chemotherapy regimens in shared wards, inadequate supportive care due to financial constraints and suboptimal patient adherence to treatment due to nonavailability of cancer hospitals in remote areas to address treatment-related complications.

Challenges encountered were many due to diverse patient demographics and modification in treatment protocols as per patient’s performance status, complicating comprehensive analysis.

Due to financial constraints, access to critical risk factors – such as genetic and molecular profiling and minimal residual disease (MRD) assessment – was limited, resulting in insufficient data for comprehensive analysis.

Despite the aforementioned limitations, this study contributes meaningful insights into the implementation and effectiveness of pediatric-inspired treatment protocols for AYA patients with acute lymphoblastic leukaemia/lymphoma in resource-limited settings. In particular, it highlights the potential feasibility and clinical impact of adapting such protocols within the Indian healthcare system, where challenges such as financial constraints, limited access to advanced diagnostics and infrastructure limitations are common. These findings underscore the importance of context-specific treatment strategies and may inform future efforts to optimise care for AYA patients in similar low- and middle-income countries.

## Conflicts of interest

None.

## Funding

Nil.

## Author contributions

Alpika Shukla – data collection, concept of study and proof reading.

Shailendra Verma – concept of study and review of literature.

Anil Tripathi – review of literature and revision of first draft of manuscript.

Swasti Sinha – design of study.

## Figures and Tables

**Figure 1.**
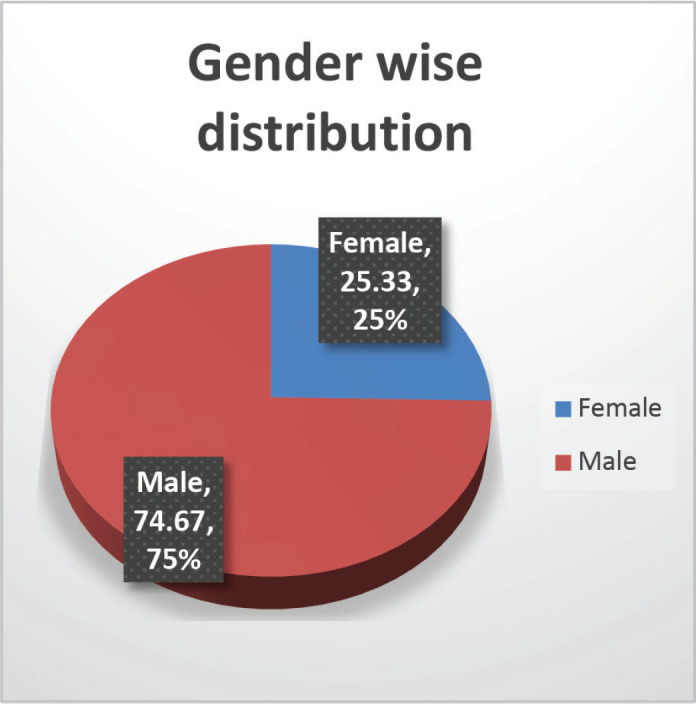
Gender-wise distribution.

**Table 1.** Baseline parameters.

Parameters	Frequency (*N*)	Percentage (%)
Gender		
Female	19	25.33
Male	56	74.67
Risk stratification		
High risk	15	20.0
Medium risk	60	80.0
PH status		
Negative	63	84.0
Positive	12	16.0
Type of acute Leukaemia		
B – ALL	52	69.33
T – ALL	23	30.67
CNS status		
CNS 1	72	96.0
CNS 3	3	4.0

**Table 2.** Toxicities.

Toxicities	Frequency	Percent
Acute kidney injury (AKI)	1	1.8
Cerebral vein thrombosis (CVT)	4	7.0
Deep venous thrombosis (DVT)	5	8.8
Febrile neutropenia (FN)	21	36.8
Fungal infection (FN)	7	12.3
Hyperglycaemia (HYG)	6	10.5
Posterior reversible encephalopathy syndrome (PRES)	1	1.8
Transaminitis (TA)	12	21.1
Total	57	100.0

**Table 3.** Distribution of study population as per outcome.

Outcome	Frequency	Percent
Disease free	33	44.0
Relapse	42	56.0
Total	75	100

## Supplementary section

### Risk stratification

Patients were categorised into three risk groups based on their clinical and genetic profiles [5].

### High risk

- Poor prednisone response and/or
- No complete remission on day 33, and/or
- Presence of high-risk cytogenetics such as:
  - t (9;22) (BCR/ABL)
  - t (4;11) (MLL/AF4)

### Moderate risk

- No high-risk criteria, and
- Initial white blood cell (WBC) count of 20 × 10⁹/l or more and/or
- T-ALL

### Standard risk

- No high-Risk criteria, and
- Initial WBC count less than 20 × 10⁹/l, and
- No T-ALL

### Treatment protocol details

The modified BFM 95 protocol included the following treatment phases and agents:

### Induction phase A (Days 1–33)

- **Tablet prednisolone**: 60 mg/m^2^ daily (Day 1–27), then tapered and stopped.
- **Vincristine (VCR)**: 1.5 mg/m^2^ IV push (max dose 2 mg) (Day 8, 15, 22, 29.)
- **Daunorubicin**: 30 mg/m^2^ IV push Day (8, 15, 22, 29)
- **L-asparaginase**: 5,000 U/m^2^ IV over 1 hour (Day 12, 15, 18, 21, 24, 27, 30, 33).

L-asparaginase was not given if the patient started on TKI in Induction Phase A.

### Induction phase B (Days 36–64)

- **Cyclophosphamide**: 1 g/m^2^ intravenous infusion over 60 minutes (Day 36 and 64)
- **Cytosine Arabinoside**: 75 mg/m^2^ (Day38–41, 45–48, 52–55, 59–62)
- **6-Mercaptopurine (6-MP)**: 60 mg/ m^2^ once daily (Day 36–63).
- **Intrathecal Methotrexate**: 12 mg (Day 45 and59).
- **TKIs**: Dasatinib 100 mg once daily (if Philadelphia chromosome +ve)

### Extracompartment Phase (M Phase) (Days 1–56)

- **Methotrexate**: 5 g/m^2^ IV over 24 hours (Day 8, 22, 36, 50).
  - **Leucovorin**: Loading dose at 42 hours after starting methotrexate, dose 50 mg/m^2^ bolus dose followed by 15 mg/m^2^ 6 hourly for 4 doses.
- **6-MP**: 25 mg/m^2^ daily (Day1–56).
- **Intrathecal methotrexate**: 12 mg (Day 8, 22, 36, 50).
- **TKIs**: Dasatinib 100 mg once a day.

### Reintensification A (Days 1–29)

- **Dexamethasone**: 10 mg/m^2^ orally (Day 1–21, then taper and stop by day 29).
- **Vincristine**: 1.5 mg/m^2^ IV push (max dose 2 mg) (Day 8, 15, 22, 29).
- **Doxorubicin**: 30 mg/m^2^ IV over 1-hour infusion (Day 8, 15, 22, 29).
- **L-asparaginase**: 10,000 U/m^2^ deep IV over 1 hour (Days 8, 15, 22, 29).

L-asparaginase was not given to patients on TKI.

### Reintensification B (Days 1–14, 24)

- **Cyclophosphamide**: 1 g/m^2^ IV infusion over 1 hour on Day 1.
- **Cytosine Arabinoside**: 75 mg/m^2^ (Day 3–6, 10–13).
- **6-MP**: 60 mg/m^2^ orally daily.
- **Intrathecal Methotrexate**: 12 mg (Day10, 24).
- **TKIs**: Dasatinib 100 mg once daily.
- **Cranial RT prophylaxis**

18 Gy in 10 fractions – high-risk

12 Gy in 7 fractions. -Standard-risk

### Maintenance Phase (Day 1–730 from initial diagnosis)

- **6-MP**: 50 mg/m^2^/day.
- **Methotrexate**: 20 mg/m^2^ weekly.
- **TKIs**: Dasatinib 100 mg once daily. (if indicated)

### Supportive care

- **Antiviral prophylaxis**: Administered with acyclovir at a dose of 400 mg twice daily.
- **Antibacterial prophylaxis**: Cotrimoxazole was recommended at a dosage of one tablet every other day.
- **Antifungal prophylaxis**: Azole antifungals were not used unless indicated based on the patient’s clinical condition or risk factors.

### Response assessment

- **Day 33 evaluation**: Bone marrow aspiration and biopsy was done on day 33 of the BFM 95 protocol Induction A. A blast percentage of less than 5% was considered indicative of remission, leading to the initiation of the BFM 95 Induction B protocol. Patients who did not achieve remission were transitioned to alternative treatment protocols.
- **Minimal residual disease assessment**: Conducted using flow cytometry after Induction B [10]. For patients with Ph-positive ALL, MRD was further assessed using real-time quantitative PCR to detect BCR-ABL translocation. Patients who tested positive for MRD were shifted to different treatment protocols.
- **Inpatient versus day-care treatments**: Induction A and high-dose methotrexate were administered as inpatient treatments, while other treatments were managed on a day-care basis. Patients were admitted as needed for the management of febrile neutropenia and drug-related toxicity.

### Treatment related toxicities

Treatment-related toxicities included a spectrum of complications such as:

- **Febrile neutropenia**
- **Pancreatitis**
- **Seizures**
- **Transaminitis**
- **Pneumonia**
- **Posterior reversible encephalopathy syndrome**
- **Febrile neutropenia**: Patients were admitted, and diagnostic tests including blood cultures, procalcitonin and galactomannan assays were conducted. Antibiotic therapy was initiated per institutional protocols. Chemotherapy was withheld until resolution of fever and recovery of blood counts.
- **Pneumonia**: Suspected cases underwent high-resolution computed tomography of the thorax. Antifungal treatment was initiated based on radiological or biochemical evidence. Bronchoalveolar lavage was performed if necessary to identify the infection’s etiology.
- Rest of the complications were less common and were managed symptomatically,

L-asparaginase was stopped in patient having seizure, chemotherapy dose was modified or withheld depending on LFT till improvement.
